# Myeloid and T Cell-Derived TNF Protects against Central Nervous System Tuberculosis

**DOI:** 10.3389/fimmu.2017.00180

**Published:** 2017-02-23

**Authors:** Nai-Jen Hsu, Ngiambudulu M. Francisco, Roanne Keeton, Nasiema Allie, Valérie F. J. Quesniaux, Bernhard Ryffel, Muazzam Jacobs

**Affiliations:** ^1^Division of Immunology, Department of Pathology, Institute of Infectious Disease and Molecular Medicine, Faculty of Health Sciences, University of Cape Town, Cape Town, South Africa; ^2^CNRS UMR7355, Experimental and Molecular Immunology and Neurogenetics, Orleans, France; ^3^South African Medical Research Council, Cape Town, South Africa; ^4^National Health Laboratory Service, Johannesburg, South Africa

**Keywords:** *Mycobacterium tuberculosis*, tumor necrosis factor, myeloid, T cell, CNS

## Abstract

Tuberculosis of the central nervous system (CNS-TB) is a devastating complication of tuberculosis, and tumor necrosis factor (TNF) is crucial for innate immunity and controlling the infection. TNF is produced by many cell types upon activation, in particularly the myeloid and T cells during neuroinflammation. Here we used mice with TNF ablation targeted to myeloid and T cell (MT-TNF^−/−^) to assess the contribution of myeloid and T cell-derived TNF in immune responses during CNS-TB. These mice exhibited impaired innate immunity and high susceptibility to cerebral *Mycobacterium tuberculosis* infection, a similar phenotype to complete TNF-deficient mice. Further, MT-TNF^−/−^ mice were not able to control T cell responses and cytokine/chemokine production. Thus, our data suggested that collective TNF production by both myeloid and T cells are required to provide overall protective immunity against CNS-TB infection.

## Introduction

Tumor necrosis factor (TNF) is a pleiotropic cytokine involved in protective cell-mediated immunity against mycobacterial infection. In humans, primates and mice, TNF is shown to be essential for the maintenance of granuloma structures and for effective control of pulmonary mycobacterial replication (1–4). Mutation of the leukotriene A4 hydrolase locus, which controls the balance of pro- and anti-inflammatory eicosanoids, showed two distinct molecular routes to mycobacterial susceptibility converging on dysregulated levels of TNF (5, 6).

Tuberculosis of the central nervous system (CNS-TB) is a severe form of extrapulmonary tuberculosis, generally caused by hematogenous dissemination of *Mycobacterium tuberculosis* (*M. tuberculosis*) after primary lung infection (7, 8). Earlier animal models of CNS-TB enabled investigation of therapeutic strategies and understanding of immune regulation during CNS-TB despite the limitation in the route of pathogen entry to the CNS (9–11). In animal studies, similar cytokine profile was observed as in CNS-TB patients (12, 13), and the genetic deficiencies of immune effectors such as TNF and iNOS have confirmed its importance as critical for immune protection against tuberculosis in the CNS (14, 15).

Homeostasis is crucial for neural activities; therefore, the interactions between the CNS and immune systems must be tightly regulated to maintain balance between immune and neural functions. This is supported by the blood–brain barrier which separates the CNS from the regular systemic blood circulation that protects the CNS from the milieu of the periphery. However, research findings have demonstrated that both resident and infiltrating immune cells participate actively in immune responses in the CNS, which upon activation may secrete cytokines including TNF (16–20). Also, using animal models of TNF deficient or overexpression, we and others have directly demonstrated the important role of TNF in pathogenesis and protective immunity during CNS-TB infection (15, 21).

In the brain, TNF is synthesized by both immune (including infiltrating cells) and non-immune cells (including neurons). Microglial cells, as the resident immune cells of the CNS, are thought to be the first line of defense and main source of TNF during CNS-TB (18, 22–24). The peripheral T cells have the ability to produce TNF along with interferon-γ (IFN-γ) that acts in both innate and specific cell-mediated immunities during mycobacterial infection (25–27). Moreover, intracerebral BCG infection induces infiltration of IFN-γ and TNF producing T cells into the CNS (18, 28). In a previous study, we reported on the prominence of TNF-mediated innate immunity during cerebral tuberculosis while neuron-derived TNF was found to be dispensable (15). Therefore, further identifying the cellular sources of TNF that contribute to the pathogenesis and immunity in the context of CNS-TB is of significant interest. In this study we investigated the functions of TNF produced collectively by myeloid and T cells in mediating immune protection against cerebral *M. tuberculosis* infection. We used mice deficient for both myeloid- and T cell-derived TNF (MT-TNF^−/−^) and investigated outcomes after intracerebral *M. tuberculosis* infection in comparative studies with TNF floxed wild-type (TNF^f/f^) and complete TNF-deficient (TNF^−/−^) mice. We demonstrate the significance of TNF production by myeloid and T cells for controlling CNS-TB infection.

## Materials and Methods

### Mice

All mouse strains, including TNF floxed wild type (TNF^f/f^), myeloid TNF deficient (MN-TNF^−/−^), T-cell TNF deficient (T-TNF^−/−^), myeloid and T-cell TNF deficient (MT-TNF^−/−^), and complete TNF deficient (TNF^−/−^) were previously described (4, 15, 19, 29) and maintained under specific pathogen-free conditions at the research animal facility of the University of Cape Town. Adult mice were PCR genotyped and used between 6 and 12 weeks of age. Infected mice were maintained under biosafety level 3 conditions. All animal procedures were approved by the Animal Research Ethics Committee, University of Cape Town, in accordance with the South African National Standard.

### Intracerebral Infection

*Mycobacterium tuberculosis* strain H37Rv was grown at 37°C in Middlebrook 7H9 broth containing 10% OADC and 0.5% Tween-80 until log phase, then aliquoted and stored at −80°C. A frozen aliquot was thawed, passed 30 times through 29 gauge needle and diluted in sterile saline. Intracerebral infection was performed using a stereotaxic approach of directly injecting H37Rv into the cerebral cortex. Prior to inoculation, a small burr hole was constructed anterior to the bregma and to the left of the midline in the skull exposing the dura mater. Mice were inoculated intracerebrally with 1 × 10^4^–1 × 10^5^ colony forming units (CFUs) of *M. tuberculosis* H37Rv using Hamilton syringe (Gastight no. 1701, Switzerland). The burr hole was sealed with bone wax and the skin sutured.

### Colony Enumeration Assay

Bacterial burdens in the brains, lungs, and spleens of infected mice were determined at specific time points after infection with *M. tuberculosis*. Organs were weighed and homogenized in 0.04% Tween 80 saline. Also, 10-fold serial dilutions of organ homogenates were plated in duplicates on Middlebrook 7H10 (Becton, Dickinson and Company) agar containing 10% OADC (Life Technologies, Gaitherburg, MD, USA), and incubated at 37°C for 19–21 days. The concentration of *M. tuberculosis* was then determined by counting the CFUs.

### Flow Cytometry

Mouse brains were collected to generate single cell suspensions. Non-specific binding to cells was blocked through incubation with αFcγRIII (1 mg/ml of rat α-mouse CD32/16c). The following antibodies were used to stain the surface markers: CD11b-PerCP-Cy5-5 (Clone M1/70), CD11c-Alexa 700 (Clone HL3), CD45-APC (Clone 30- F11), CD80-FITC (16–10 A1), CD86-V450 (Clone GL1), MHCII/(I-A/I-E)-PE (M5/114.15.2), CD3?-Pacific Blue, CD4-Alexa 700, and CD8-PerCP-Cy5.5. All antibodies were from BD Pharmingen™ and BD Horizon™. After staining, cells were washed and fixed, then analyzed on BD LSR Fortessa (Beckton Dickinson) flow cytometer using Cell Quest software.

### Quantification of Chemokines and Cytokines

Supernatants from brain homogenates were prepared for cytokine and chemokine measurement by enzyme-linked immunosorbent assay (ELISA) after 3 weeks subsequent to intracerebral *M. tuberculosis* infection. The chemokines MCP-1, MIP-1α, and RANTES; and the cytokines IFN-γ, IL-2, IL-6, IL-12p70, and TNF (R&D Systems, Germany) were measured using commercially available ELISA reagents according to the manufacturer’s instructions. Chemokine and cytokine concentrations were measured by absorbance using a Versamax Microplate Reader (Molecular Devices, LLC, CA, USA) with SoftMax software.

### Statistical Analysis

The data are presented as the mean ± SEM. Statistical analysis was performed by two-way ANOVA and one-tailed *t*-test. For all tests, a *p*-value of ≤0.05 was considered significant.

## Results

### TNF Produced by Myeloid Cell and T-Cell Are Required for Protection against *M. tuberculosis* Infection in the Brain (Similar to TNF^−/−^)

**Figure 1:**
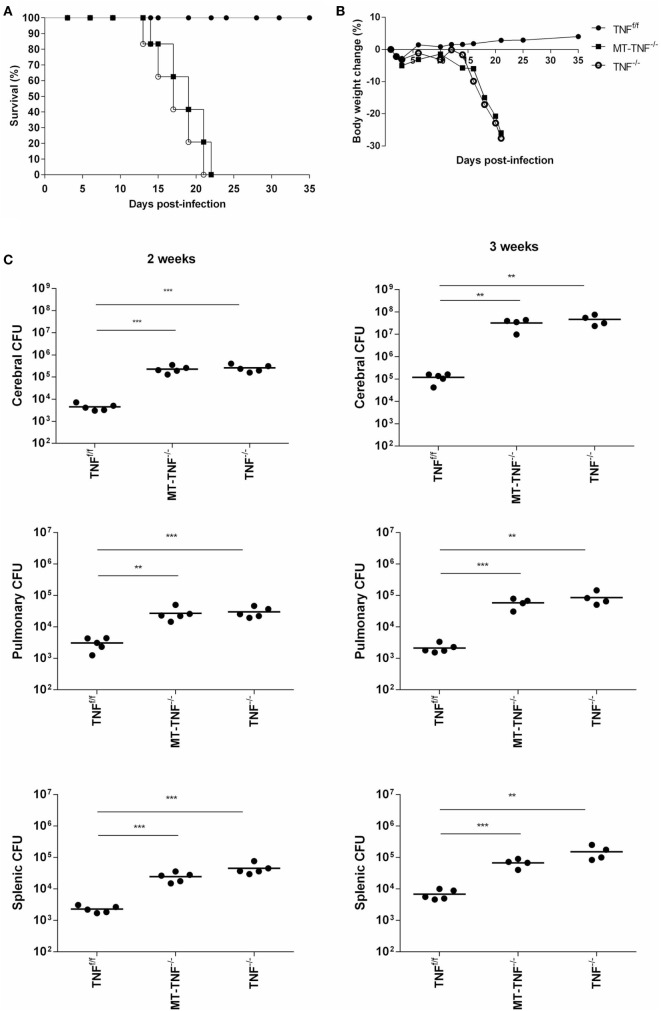
**Tumor necrosis factor (TNF) produced by myeloid and T-cells is essential for protection against *Mycobacterium tuberculosis* infection in the brain**. TNF^f/f^ (black circle), MT-TNF^−/−^ (black square) and TNF^−/−^ (clear circle) mice were infected with *M. tuberculosis* H37Rv at a dose of 1 × 10^4^–1 × 10^5^ CFU/brain by intracerebral inoculation. Mortality **(A)** and body weight changes **(B)** were measured and recorded for the experimental duration (*n* = 5–7 mice/group). **(C)** Bacterial burdens in brains, spleens, and lungs were determined at day 14 and 21 postinfection. These data were one representative out of three to five independent experiments (*p ≤ 0.05, **p ≤ 0.01, and ***p ≤ 0.001).

Myeloid and T-cells contribute significantly to the pathogenesis of pulmonary tuberculosis (4, 30). To address the contribution of TNF derived from myeloid and T cells in protective immunity against CNS-TB, we intracerebrally challenged the mice with *M. tuberculosis* and compared disease progression in various cell-specific TNF-deficient mice, including myeloid specific (MN-TNF^−/−^), T-cell specific (T-TNF^−/−^), and TNF deficient in both myeloid and T-cells (MT-TNF^−/−^). We found that MN-TNF^−/−^ and T-TNF^−/−^ mice survived the infection similar to wild-type TNF^f/f^ mice (Table 1); therefore, TNF deficiency in either myeloid cells or T cells had no effects on the overall protection against cerebral tuberculosis. Interestingly, the MT-TNF^−/−^ mice succumbed to the infection similar to the complete-deficient TNF^−/−^ mice (Table 1; Figures 1A,B). As previously reported (15), TNF^−/−^ mice were highly susceptible to cerebral *M. tuberculosis* infection resulting in rapid weight loss and death by 3 weeks postinfection. In comparison to TNF^−/−^ mice, MT-TNF^−/−^ mice were similarly highly susceptible and rapidly succumbed to infection with >20% body weight loss (Figures 1A,B).

**Table 1:** **Clinical parameters of cerebral tuberculosis in various cell-specific tumor necrosis factor (TNF)-deficient mice**.

	Day 25		Day 105	
	Mortality (%)	Body weight change (%)	Mortality (%)	Body weight change (%)
TNF^f/f^	0 (0/27)	2.8	0 (0/27)	4.6
MN-TNF^−/−^	0 (0/20)	1.9	0 (0/20)	2.9
T-TNF^−/−^	0 (0/22)	1.3	4.5 (1/22)	3.4
MT-TNF^−/−^	94.1 (16/17)	−26.8	–	–
TNF^−/−^	91.7 (22/24)	−21.4	–	–

*The data are pooled of at least three independent experiments*.

To further investigate the synergistic role of myeloid and T-cell-derived TNF to control cerebral bacilli replication, we assessed bacterial burden in the brains of *M. tuberculosis* infected TNF^f/f^, MT-TNF^−/−^, and TNF^−/−^ mice (Figure 1C). MT-TNF^−/−^ and TNF^−/−^ mice exhibited similar significant increases in cerebral bacilli burdens compared to TNF^f/f^ mice at day 14 (*p* < 0.001) and day 21 (*p* < 0.01) postinfection. We next assessed the extent of dissemination of bacilli from the brain by measuring the levels of pulmonary and splenic bacterial burden (Figure 1C). MT-TNF^−/−^ mice showed mycobacterial dissemination similar to the TNF^−/−^ mice in both lungs and spleens which were 1–2 log_10_ higher than TNF^f/f^ wild-type mice. Thus, these data demonstrate that myeloid and T cell-derived TNF is a collective requirement for cerebral immune protection and control of *M. tuberculosis* replication.

### Myeloid Cell and T-Cell-Derived TNF Are Essential to Control Pathology and Inflammation during Cerebral Tuberculosis

**Figure 2:**
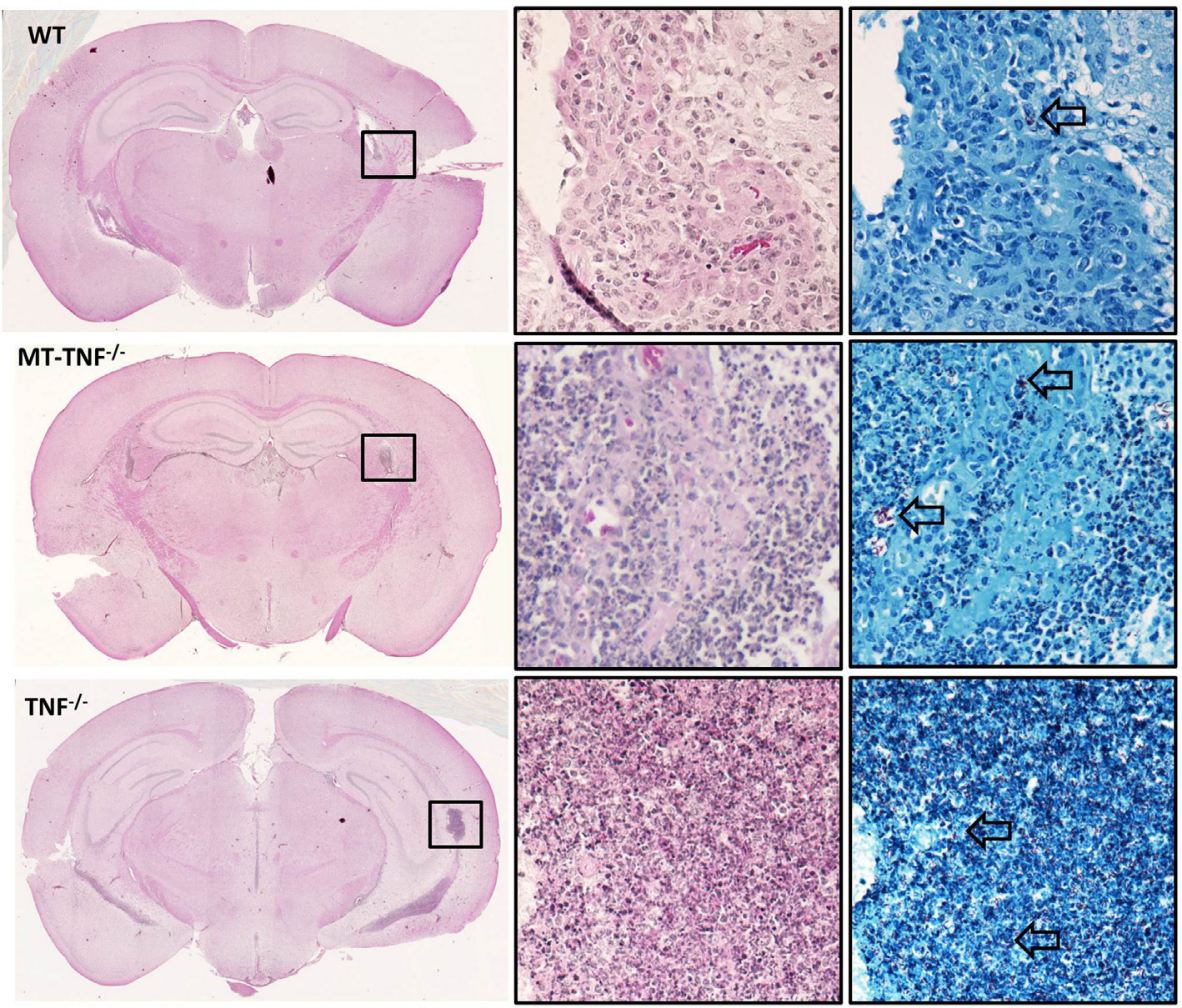
**Myeloid cells and T-cells are important source for tumor necrosis factor (TNF)-dependent pathology during cerebral tuberculosis**. Hematoxylin and eosin-stained coronal brain sections of TNF^f/f^, MT-TNF^−/−^, and TNF^−/−^ mice (*n* = 5 mice/group) at day 21 postinfection are presented. Ventriculitis of the choroid plexus accompanied by necrosis (box) are observed. Corresponding Ziehl–Neelsen stain revealed acid-fast bacilli (arrows). The images are representatives of three to five independent experiments.

We next examined the synergistic role of myeloid and T-cell-derived TNF to control cerebral inflammation by analysis of brain histopathology in *M. tuberculosis*-infected mice at 3 weeks postinfection (Figure 2). In the brain sections taken from TNF^f/f^ and TNF^−/−^ mice, we observed similar degrees of inflammation as to what we reported previously (15). The cerebral inflammation was controlled in TNF^f/f^ mice, suggested by the predominant lymphocytic infiltration found in the choroid plexus and ventricles of TNF^f/f^ brains. In contrast, both MT-TNF^−/−^ and TNF^−/−^ mice exhibited severe neuropathology and uncontrolled inflammation where cellular infiltration was extended to the periventricular tissue. Similar to TNF^−/−^ mice, MT-TNF^−/−^ mice also had foci of necrosis in the brain tissue where acid fast bacilli were largely found.

**Figure 3:**
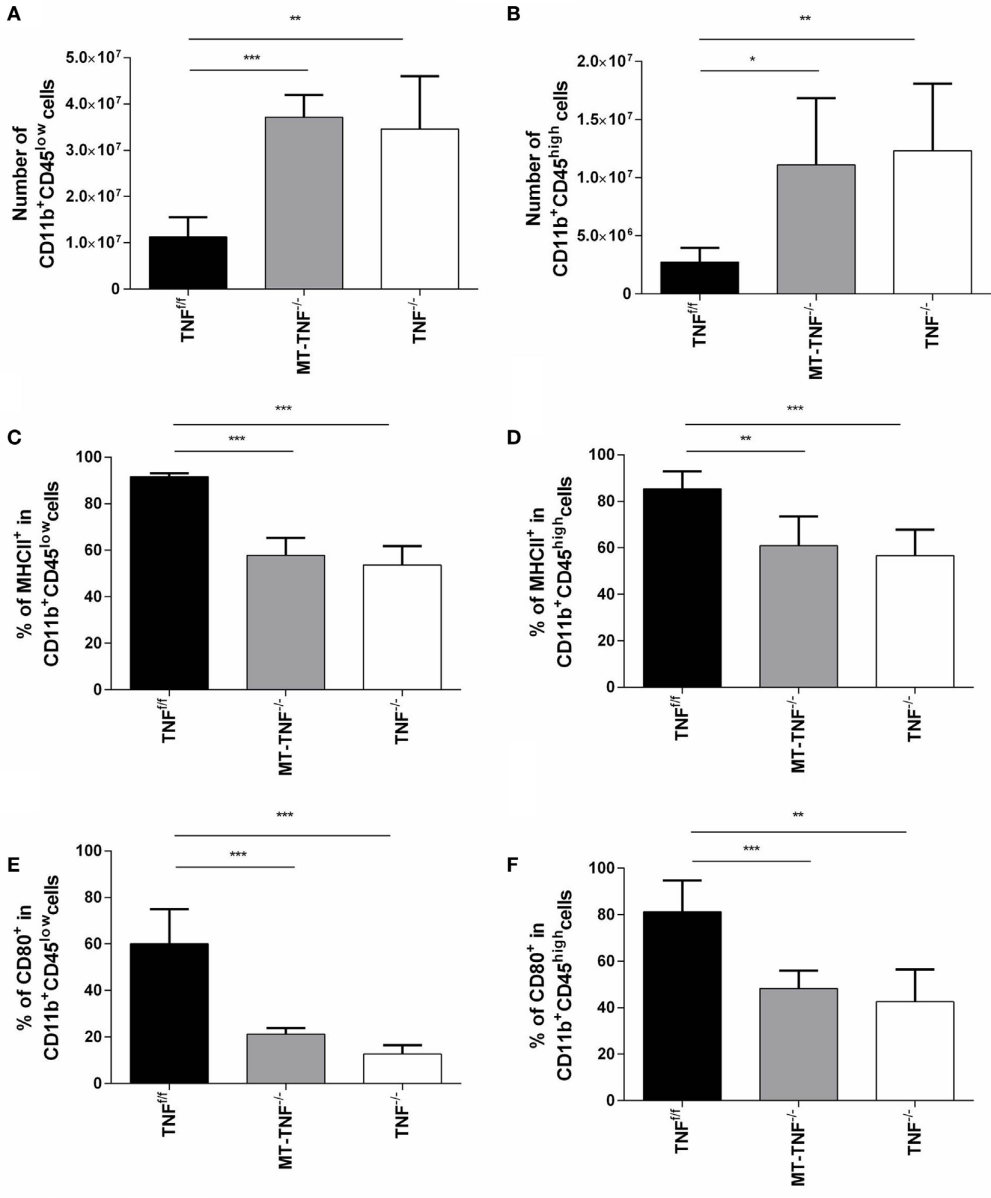
**Tumor necrosis factor (TNF) production by myeloid cells and T-cells is required for activation and regulation of innate immunity during cerebral tuberculosis**. TNF^f/f^ (black), MT-TNF^−/−^ (gray), and TNF^−/−^ (clear) mice were infected with *Mycobacterium tuberculosis* H37Rv at a dose of 1 × 10^4^–1 × 10^5^ CFU/brain by intracerebral inoculation. The number of **(A)** CD11b^+^CD45^low^ microglia and **(B)** CD11b^+^CD45^high^ macrophages and the frequency of **(C,D)** MHC II and **(E,F)** CD80 expression was analyzed by flow cytometry at day 21 postinfection. Data (mean ± SEM of five mice) are representative of three repeat experiments (*p ≤ 0.05, **p ≤ 0.01, and ***p ≤ 0.001).

The neuropathology observed in the MT-TNF^−/−^ mice suggested defective TNF-mediated cellular recruitment and innate immune responses in response to *M. tuberculosis* challenge. We, therefore, quantified microglia (CD11b^+^CD45^low^) and macrophages (CD11b^+^CD45^high^) in the brain and evaluated activation status of these innate immune cells subsequent to 3 weeks cerebral *M. tuberculosis* infection. The flowcytometric analysis revealed significantly higher (*p* < 0.05) frequencies and absolute cell numbers of microglia and macrophages in both TNF^−/−^ and MT-TNF^−/−^ mice (Figure 3). To investigate the capacity of microglia and macrophages to present antigen and activate lymphocytes, we analyzed the expression of MHCII, CD80, and CD86. Apart from similar levels of CD86 in all three mouse strains (data not shown), we observed significantly lower (*p* < 0.01) expression of CD80 and MHCII in MT-TNF^−/−^ compared to TNF^f/f^ mice (Figure 3).

Therefore, myeloid cell and T-cell-derived TNF were required to regulate CNS inflammation during *M. tuberculosis* infection; moreover, it determined the quality of the innate immune response.

### Absence of TNF on Myeloid and T Cell Renders Defective T-Cell Responses during Cerebral Tuberculosis

**Figure 4:**
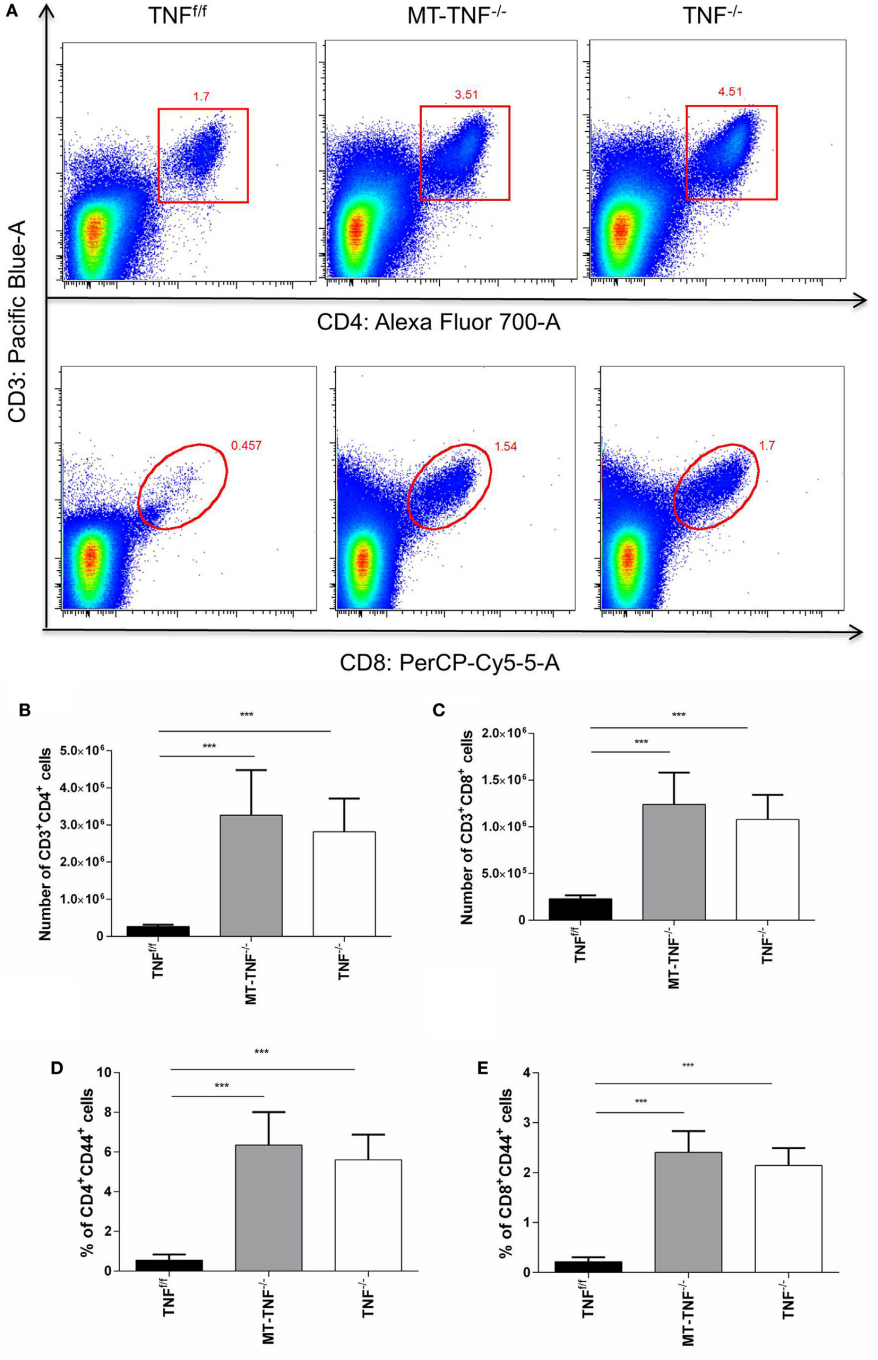
**Cerebral tuberculosis-induced T-cell response is dependent on the myeloid and T-cells-derived tumor necrosis factor (TNF)**. **(A)** Representative FACS plots of CD3^+^CD4^+^ and CD3^+^CD8^+^ cells in TNF^f/f^, MT-TNF^−/−^, and TNF^−/−^ mice at day 21 after intracerebrally infected with *Mycobacterium tuberculosis* H37Rv. The number of **(B)** CD3^+^CD4^+^ and **(C)** CD3^+^CD8^+^, and the frequency of **(D)** CD4^+^CD44^+^ and **(E)** CD8^+^CD44^+^ expression, was analyzed by flow cytometry at day 21 postinfection. Data (mean ± SEM of five mice) are representative of three repeat experiments (*p ≤ 0.05, **p ≤ 0.01, and ***p ≤ 0.001).

To explore whether the myeloid- and T-cell-derived TNF alter influx of T cells into the brain, we quantified CD3^+^CD4^+^ and CD3^+^CD8^+^ T cell recruitment in the TNF^f/f^, MT-TNF^−/−^, and TNF^−/−^ mouse brains after *M. tuberculosis* cerebral infection. We observed an overall increase in the frequencies of CD3^+^CD4^+^ and CD3^+^CD8^+^ T cells in the brains of all strains over 3 weeks postinfection, especially more in MT-TNF^−/−^ and TNF^−/−^ at all time points (data not shown). At 3 weeks postinfection, the frequency of CD3^+^CD4^+^ cells in the MT-TNF^−/−^ and TNF^−/−^ mouse brains was more than twofold higher compared to TNF^f/f^ mice (Figure 4A). The frequencies of CD3^+^CD8^+^ T cells in MT-TNF^−/−^ and TNF^−/−^ mice at week 3 postinfection were found more than threefold higher compared with TNF^f/f^ mice. Comparatively, MT-TNF^−/−^ and TNF^−/−^ mice exhibited similar frequencies of CD3^+^CD4^+^ and CD3^+^CD8^+^ T cells in the brain (Figure 4A). When assessing the absolute cell numbers of infiltrating cells at 3 weeks postinfection, we found equivalent numbers of CD3^+^CD4^+^ (Figure 4B) and CD3^+^CD8^+^ (Figure 4C) T cells in the MT-TNF^−/−^ and TNF^−/−^ mice, which were significantly higher compared to TNF^f/f^ mice. To further examine the effector function of these T cells, we analyzed the expression levels of CD44. The percentage of CD3^+^CD4^+^ (Figure 4D) and CD3^+^CD8^+^ (Figure 4E) T cells expressing CD44 in both MT-TNF^−/−^ and TNF^−/−^ mice were significantly higher (*p* < 0.001) than in TNF^f/f^ mice at week 3 postinfection. Therefore, these data suggest that in the absence of myeloid and T cell-derived TNF, as well as in the complete ablation of TNF, the overall T cell response was deregulated by means of T cell influx and activation.

### Differential Regulation of Cytokines and Chemokines by Myeloid and T-Cell-Derived TNF during Cerebral *M. tuberculosis* Infection

**Figure 5:**
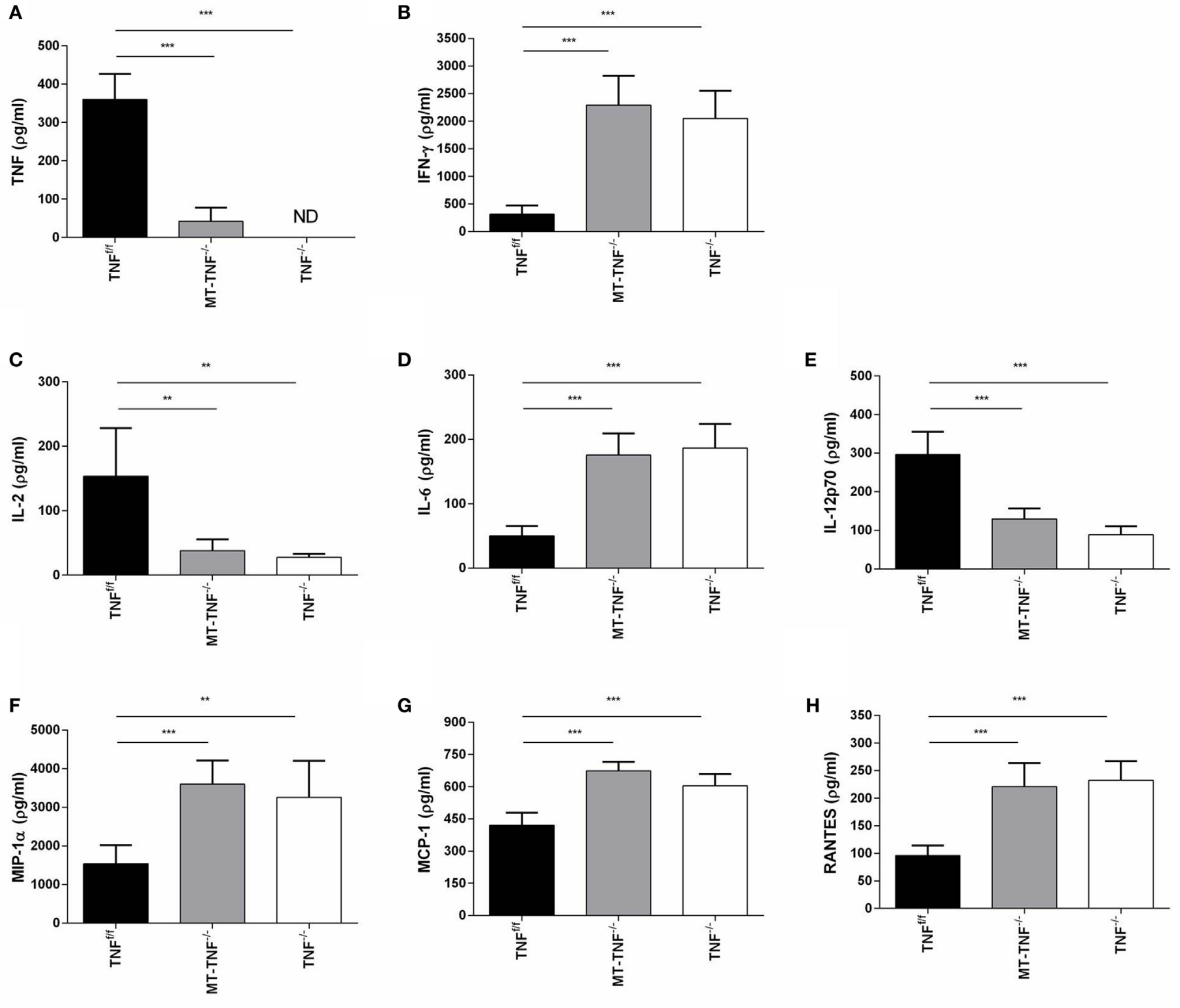
**Myeloid and T-cells-derived tumor necrosis factor (TNF) regulates synthesis of cytokine and chemokines**. **(A)** TNF, **(B)** IFN-γ, **(C)** IL-2, **(D)** IL-6, **(E)** IL-12p70, **(F)** MIP-1α, **(G)** MCP-1, and **(H)** RANTES cerebral concentrations were measured by ELISA in TNF^f/f^, MT-TNF^−/−^, and TNF^−/−^ mice at day 21 after intracerebrally infected with *Mycobacterium tuberculosis* H37Rv. Data (mean ± SEM of five mice) are representative of at least three repeat experiments (*p ≤ 0.05, **p ≤ 0.01, and ***p ≤ 0.001).

To further dissect the mechanism behind the increased innate immune cell recruitment and T cell development, we analyzed the levels of cytokines and chemokines induction in response to *M. tuberculosis* infection. We measured concentrations of the cytokines, TNF, IFN-γ, IL-2, IL-6, and IL-12p70, as well as the chemokines, MIP-1α, MCP-1, and RANTES, in the brain homogenates of TNF^f/f^, MT-TNF^−/−^, and TNF^−/−^ mice after 3 weeks intracerebral infection (Figure 5).

As expected, no TNF production was detected in TNF^−/−^ mice and the induction of TNF in TNF^f/f^ mice confirmed its requirement as part of protective immune responses. TNF expression in MT-TNF^−/−^ mice indicated that TNF in the 3 weeks *M. tuberculosis*-infected brain were mostly contributed by myeloid and T cells. Similar to the reduced TNF production of MT-TNF^−/−^ mice, IL-2 and IL-12p70 in MT-TNF^−/−^ mouse brains were also significantly lower compared to TNF^f/f^ mice. Alternatively, we observed increased levels of IFN-γ and IL-6 production in MT-TNF^−/−^ mice, which were significantly higher than the TNF^f/f^ mice. We found similar expression levels in both MT-TNF^−/−^ and TNF^−/−^ infected brains, which were significantly higher (*p* < 0.01) than the TNF^f/f^-infected brains.

Therefore, data showed that myeloid and T cell-derived TNF have differential effects on cytokine and chemokine production during cerebral *M. tuberculosis* infection. These results also illustrate that deletion of TNF from myeloid and T cells rendered an uncontrolled increase of IFN-γ and IL-6 protein and reduced IL-2 and IL-12p70 production. The results further demonstrate that ablation of TNF in myeloid and T cells resulted in an impaired chemoattractant response during cerebral *M. tuberculosis* infection.

## Discussion

It has been demonstrated that elevated levels of TNF are associated with TB meningitis and tuberculoma (12, 13). More recently, data from human, mouse, and zebrafish indicate that a relative deficiency of TNF results in destructive immunopathology during CNS-TB infection (6, 15). This study has shown that TNF is absolutely required for protective host immune responses during CNS-TB and is in agreement with the previous reports. In the CNS, TNF is produced by immune and non-immune cells (20); however, the mechanisms by which cell-specific TNF acts to control CNS-TB and cellular recruitment to the infection site are not clearly defined. In this study, MT-TNF^−/−^ mice were highly susceptible to cerebral tuberculosis infection. Therefore, we have demonstrated ablation of TNF from myeloid and T cells resembled complete ablation of TNF, suggesting that myeloid and T cells are major sources of TNF in mediating immune protection against CNS-TB. It also provides important evidence that synergistic activity of TNF derived from myeloid cells and T cells is required for optimum protection.

As part of the host–pathogen interaction, *M. tuberculosis* induces a TNF-dependent host immune response orchestrated from various cell types (4, 24, 31, 32). TNF signaling through TNF receptors (TNFRp55 and TNFRp75) forms an integral component in protective immunity against tuberculosis (1, 30, 33). To dissect the cellular contribution to TNF functions, Grivennikov and colleagues were first to generate and report the cell-specific TNF-deficient mice. They used Cre-loxP recombination system to target TNF gene and generated TNF^f/f^ and TNF^−/−^ mouse together with a panel of cell-specific TNF-deficient mice and successfully demonstrated selective and efficient ablation of TNF in myeloid cells or/and lymphocytes of these mice by ELISA and intracellular TNF staining (29). The use of cell-specific gene-deficient mice has yielded insight into regulatory roles of cell type-specific expression of TNF and TNFRp55 in immune control during mycobacterial infection (4, 15, 29, 30). Previously, we have identified myeloid and lymphoid cells as critical sources of TNF for control of pulmonary *M. tuberculosis* infection; while myeloid TNF is required for early innate immune protection, T cell-derived TNF has a prominent role for chronic infection. Moreover, myeloid and T cell TNF act collectively in pulmonary tuberculosis resembling the effect of overall TNF (4). This distinct cellular contribution of TNF has also been seen in neuroinflammation. Kruglov and colleagues have shown that myeloid and T cells are the critical source of TNF during EAE, where the MT-TNF^−/−^ mice recapitulated the pathology and disease progression seen in complete TNF-deficient mice (19). Despite the different pathogenesis in EAE and CNS-TB, our current study suggested that myeloid cells and T cell are the main source of total TNF production in the brain at day 21 postinfection. Although, there are differences in organ-specific makeup between the brain and lung, the overall results of MT-TNF^−/−^ mice in CNS-TB are consistent with those seen in pulmonary TB (4), indicating that the underlying immune protective mechanisms in both pulmonary and CNS-TB model are similar.

During pulmonary *M. tuberculosis* infection, the innate myeloid cells are crucial for immune protection in a TNF/TNFRp55-dependent manner. The absence of TNF or TNFRp55 in macrophages and dendritic cells dramatically impaired the host responses to acute pulmonary *M. tuberculosis* infection (4, 30). In the CNS, microglia are considered the resident macrophages and shared the same myeloid origin as the peripheral monocytes during early development; however, emerging evidence suggests that microglia differ considerably from the circulating macrophages (34, 35). Some concerns have been raised with respect to the use of Cre-LysM system to target gene deletion specifically in microglia in which Cre transgene is constitutively expressed in the lysozyme loci of myeloid cells. Different data were reported on the percentage of gene recombination/deletion in microglia (36–38); however, it was shown that the difference in efficiency of gene recombination lies in the active states of microglia (38). In this study, we have analyzed the numbers and the functionality of CD11b^+^CD45^low^ and CD11b^+^CD45^high^ cells; however, it is not within the scope of our study to differentiate the TNF contribution from resting to active microglia. We have shown the ability of myeloid-specific TNF-deficient mice to survive the cerebral *M. tuberculosis* infection; however, we do not discriminate the important role of microglia as the main source of TNF in the brain during CNS-TB (18, 22–24). Although we did not observe mortality in the MN-TNF^−/−^ mice after cerebral *M. tuberculosis* infection, it is likely that TNF production from other cell-types compensate the effects (4, 18, 20). To gain more insight on TNF production by other cell types in the CNS, we have recently generated neuron-specific TNF-deficient mice (Ns-TNF^−/−^) to study the role of neuron-derived TNF in CNS-TB (15). In our previous report, we have demonstrated specific TNF ablation in the neurons of Ns-TNF^−/−^ mouse by intracellular TNF staining that shows TNF production in the TNF^f/f^ neurons, but not in the Ns-TNF^−/−^ and TNF^−/−^ neurons. While neuron-derived TNF is dispensable in protective immunity against CNS-TB, neurons contribute to TNF production during CNS-TB (15). Astrocytes as the most abundant glial cell population are also capable of secreting TNF in response to various stimuli (39–41). Although astrocytes have been shown to internalize *M. tuberculosis* bacilli (23), the roles of reactive astrocytes during the response to *M. tuberculosis* infection are only beginning to be elucidated.

Unlike the MN-TNF^−/−^ mice, uncontrolled cellular recruitment and poor immune activation in MT-TNF^−/−^ mice have provided conclusive evidence that collective TNF production by myeloid and T cells is critical in immune regulation during CNS-TB infection. Taken together the survival of MN-TNF^−/−^ and T-TNF^−/−^ mice in CNS-TB, we also speculate the synergistic effect of myeloid and T cell-derived TNF in chronic infection. It has been demonstrated in a chimeric transfer study that both macrophage and T cell-derived TNF are required for sufficient and long-term protection against *M. tuberculosis* infection (26). The control of latent infection is TNF dependent that requires macrophage and T cells to form and maintain the granuloma structure (32, 42, 43). Infected macrophages produce TNF and induce the expression of chemokines, such as IL-8, MCP-1, and RANTES, which provide signals for migration of immune cells to the sites *of M. tuberculosis* infection (44, 45). As evident in our study, we found deregulated expression of chemokines in the absence of TNF expression in myeloid and T cells that causes a profound effect on the cellular recruitment.

Tumor necrosis factor is involved in both immune and immunomodulatory responses and acts in synergy with T cell-derived IFN-γ to enhance the macrophages antimycobactericidal activity of nitric oxide and other RNI by iNOS. IFN-γ is mainly produced by T cells and natural killer cells in response to dying bacterial-infected macrophages (46, 47); the uncontrolled increase IFN-γ concentrations observed in the respective mouse strains correlated with the increase infiltrating of effector CD4+ and CD8+ T cells. A study by Flynn et al. (1) using IFN-γ^−/−^ mice clearly demonstrated the important role of IFN-γ in the protective immune response to *M. tuberculosis* infection, while other studies using transgenic mice have shown that IFN-γ overexpression in the CNS increases disease progression (48, 49). Our studies that show a correlation between high IFN-γ expression and advanced TB disease support these findings.

Besides increase production levels of IFN-γ, the cytokine IL-2 was found to be lower in MT-TNF^−/−^ and TNF^−/−^ brains, yet, the infiltration of effector T cells was higher than in the TNF^f/f^ mice. This coincides with the previous findings of dysregulated T cell trafficking to the brains of IL-2-deficient mice attributes to CNS autoimmunity (50–52). Moreover, the loss of brain IL-2 expression changes the neuroimmunological milieu such as an increase in concentrations of endogenous CXCL10, MCP-1, and IL-15, which is a potent T cell chemoattractant shares the same IL-2 receptor subunits for signal transduction (53–55). Originally, IL-2 described as a potent T cell growth factor is known to promote the proliferation and survival of effector T cells. Recent studies have highlighted the role of IL-2 in the regulation of immune responses and regulatory T cell (Treg) homeostasis where cytokine competition is crucial for the balance between tolerance and response (56–58). Tregs are a population of T cells characterized by the expression of CD25 (IL-2Ra) and the transcription factor Foxp3, and their general role has been demonstrated as immunosuppressive in various experimental settings of autoimmune diseases (59, 60). Another possibility of lower brain IL-2 production in MT-TNF^−/−^ and TNF^−/−^ mice may be a direct result of TNF deficiency. Although the proinflammatory effects of TNF signaling through TNFRp55 and TNFRp75 are well documented, increasing evidence indicates the immunoregulatory effects of TNF–TNFRs on Treg cell function (59, 61, 62). The expansion of Tregs upon mycobacterial infection has been shown in animal and clinical studies (63, 64). Moreover, the accumulation of Tregs was found in the CNS after mycobacterial and viral infection, which regulates T cells trafficking to CNS (18, 65). We have previously shown a modulatory role of TNFRp75 shedding-mediated TNF signaling in IL-12-dependent dendritic cell migration and *M. tuberculosis*-specific T cell activation (33). Recently, it has been shown that TNF–TNFRp75 interaction plays a critical role in the expansion of Tregs (61, 66, 67). Here, we observed an increase in chemokine production in MT-TNF^−/−^ mice and comparable to TNF^−/−^ mice, which correlated with increased recruitment of infiltrating leukocytes into the brain. Therefore, it is possible in our study that the absence of TNF disrupts Treg function and causes uncontrolled T cell responses during CNS-TB infection. Taken together, TNF production by myeloid cell and T-cell is pivotal in regulating T cell immunity.

In conclusion, our data demonstrate that ablation of TNF in myeloid and lymphoid T cells renders the protective immunity ineffective, leading to increase susceptibility. In conclusion, the findings in this study corroborate earlier reports of the importance of TNF in immune-mediated responses against tuberculosis. We further showed that the TNF production by both myeloid cell and T-cell is essential for this process and the future development of improved diagnostic and therapeutic strategies.

## Author Contributions

N-JH, NMF, RK, and NA were involved in experiments and data analysis. VQ and BR contributed to the experimental design. N-JH wrote the manuscript. MJ oversaw the project. All authors read and approved the final manuscript.

## Conflict of Interest Statement

The authors declare that the research was conducted in the absence of any commercial or financial relationships that could be construed as a potential conflict of interest.
